# Modeling and Analysis of the Noise Performance of the Capacitive Sensing Circuit with a Differential Transformer

**DOI:** 10.3390/mi10050325

**Published:** 2019-05-15

**Authors:** Yafei Xie, Ji Fan, Chun Zhao, Shitao Yan, Chenyuan Hu, Liangcheng Tu

**Affiliations:** 1 MOE Key Laboratory of Fundamental Physical Quantities Measurement & Hubei Key Laboratory of Gravitation and Quantum Physics, PGMF and School of Physics, Huazhong University of Science and Technology, Wuhan 430074, China; xieyaphe@hust.edu.cn (Y.X.); chun_zhao@hust.edu.cn (C.Z.); yanshitao@hust.edu.cn (S.Y.); chenyuanhu@hust.edu.cn (C.H.); 2 Institute of Geophysics, Huazhong University of Science and Technology, Wuhan 430074, China; 3 TianQin Research Center for Gravitational Physics and School of Physics and Astronomy, Sun Yat-sen University (Zhuhai Campus), Zhuhai 519082, China

**Keywords:** capacitive sensing, low noise circuit, differential transformer, resonant frequency, *LQ* product

## Abstract

Capacitive sensing is a key technique to measure the test mass movement with a high resolution for space-borne gravitational wave detectors, such as Laser Interferometer Space Antenna (LISA) and TianQin. The capacitance resolution requirement of TianQin is higher than that of LISA, as the arm length of TianQin is about 15 times shorter. In this paper, the transfer function and capacitance measurement noise of the circuit are modeled and analyzed. Figure-of-merits, including the product of the inductance *L* and the quality factor *Q* of the transformer, are proposed to optimize the transformer and the capacitance measurement resolution of the circuit. The *LQ* product improvement and the resonant frequency augmentation are the key factors to enhance the capacitance measurement resolution. We fabricated a transformer with a high *LQ* product over a wide frequency band. The evaluation showed that the transformer can generate a capacitance resolution of 0.11 aF/Hz^1/2^ at a resonant frequency of 200 kHz, and the amplitude of the injection wave would be 0.6 V. This result supports the potential application of the proposed transformer in space-borne gravitational wave detection and demonstrates that it could relieve the stringent requirements for other parameters in the TianQin mission.

## 1. Introduction

High-performance capacitive sensing techniques have been widely applied in many fields [1,2,3], especially in the development of the electrostatic space accelerometers in the satellite missions for Earth’s gravity recovery, such as Gravity Recovery And Climate Experiment (GRACE) and Gravity field and steady-state Ocean Circulation Explorer (GOCE) [4,5], and the space-borne gravitational experiments, such as MICROSCOPE (a space mission to test the equivalence principle) [6] and Laser Interferometer Space Antenna (LISA), a space-borne gravitational wave detection mission [7,8]. Among various displacement sensing technologies, high-performance capacitive sensing presents outstanding advantages, such as low power consumption, high integration capability, and potentially low cost. Many studies have discussed and analyzed in detail the general principles of the design of a capacitive transducer and its space applications [9,10,11,12,13,14].

Current state-of-the-art capacitive transducer measurement resolution could meet the requirements of space-based gravitational wave detection. The expected capacitance sensitivity performance, which came to be about 1 aF/Hz^1/2^ across the bandwidth of 0.1 mHz to 0.1 Hz for LISA, was discussed by Weber et al. [15]. Armano et al. [16] reported the latest results of the capacitive sensitivity of the key payload used in the LISA Pathfinder (LPF) satellites. The capacitive sensors could achieve the resolution of 0.7–1.8 aF/Hz^1/2^.

Besides LISA and other similar projects [17,18], Luo et al. [19] have also proposed the TianQin mission, a space-borne detector of gravitational waves in the millihertz frequency ranges. TianQin is planning to launch three spacecraft into the orbits around the Earth. The easily accessible geocentric orbits allow for the use of more readily available spacecraft technologies that may significantly reduce the overall cost. However, the arm length of TianQin is about 1.7 × 10^5^ km, which is almost 15 times shorter than that of LISA. This makes the arm length variation 15 times smaller than that of LISA for a given gravitational wave [19,20,21,22], resulting in higher accuracy specifications for the laser interferometer and the disturbance reduction system (DRS) of TianQin compared with those of LISA.

Table 1 summarizes the comparison of some parameters for the missions of LISA and TianQin. It can be seen that the capacitance sensitivities of these two missions are similar. However, this requirement for TianQin is a compromise between several parameters. For instance, the amplitude of injection sine voltage used in TianQin is two times higher than in LISA. Both these two missions used a gold-coated Platinum–Gold (Pt–Au) alloy cube as the test mass (TM). In order to detect the movement of the test mass, a number of capacitive sensing electrodes are positioned, surrounding the test mass. The electrostatic stiffness between the test mass and the electrodes increases in proportion to the square of the amplitude of the injection sine voltage [23]. More importantly, a high electrostatic stiffness will result in a poor acceleration sensitivity. Therefore, if a high capacitance resolution of sub-aF, which is better than the requirement of TianQin, could be achieved, the requirement of the amplitude of the injection voltage would decrease. This would also relieve the stringent requirements for other parameters [15,19,24,25]. For example, a higher capacitance resolution could result in a lighter test mass without the loss of the acceleration sensing resolution.

In this paper, we first model and analyze in details the transfer function and the capacitance measurement noise of the capacitive sensing circuit with a differential transformer, focusing on decreasing the current noise of the sensing circuit and elevating the capacitance sensitivity. We found that the *LQ* product (*L*, the transformer inductance; *Q*, the transformer quality factor) and the resonant frequency (*ω*_0_) are the key factors to enhance the capacitance resolution. We then fabricated a transformer with an excellent *LQ* parameter. Using this transformer, we could obtain the effective capacitance sensitivity of 0.11 aF/Hz^1/2^ at an optimized resonant frequency of 200 kHz, while the amplitude of the injection wave is 0.6 V. This result demonstrates the feasibility of the transformer’s potential application in space-borne gravitational wave detection in the TianQin mission.

## 2. Front-End Circuit Analysis

### 2.1. Sensing Function

**Figure 1:**
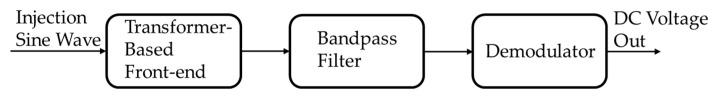
Block diagram of the capacitive sensing circuit with a differential transformer.

The bandwidths of LISA and TianQin are both in the range of a few millihertz, in which the 1/*f* noise dominates the noise level of amplifiers. Modulation–demodulation technology could bring the useful signal to a higher frequency. In this case, the effect of 1/*f* noise can be effectively eliminated. The complete sensing block diagram [26] is shown in Figure 1. With the injection wave, the capacitive sensing signal can be converted into a voltage signal by the transformer-based front-end circuit. This voltage is then band filtered at the injection frequency. After that, the filtered wave is demodulated by a synchronous demodulator, which has the same frequency source as the injection sine wave to lower the noise from the demodulation process. The final DC voltage can be further processed.

**Figure 2:**
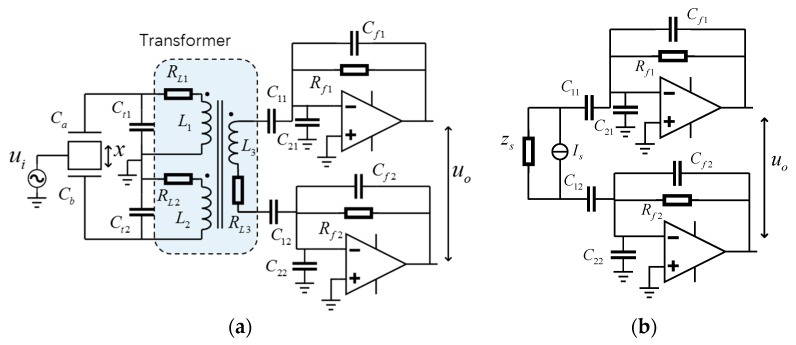
(**a**) The front-end circuit model with a differential transformer and (**b**) its equivalent circuit. *C_t_*_1_ and *C_t_*_2_ are resonant frequency tuning capacitors. They consist of both the internal stray capacitors of the transformer primary windings and the other capacitors used there. *C*_11_ and *C*_12_ are much larger than other capacitors in the circuit. Combining *C*_11_ and *C*_12_ with *R_f_*_1_ and *R_f_*_2_, the lattice is used to limit the DC gain, and thus prevents the amplifiers from saturation.

The front-end circuit of the capacitive sensing circuit with a differential transformer is shown in Figure 2a, where *C_a_* and *C_b_* are capacitors under test. If a transformer with a high performance is applied in the circuit, a better measurement resolution should be obtained.

To simplify the analysis, according to Norton’s theorem [27], the transformer network can be replaced by a current source in parallel with a reactive impedance. Then the circuit could be simplified as shown in Figure 2b. Considering ideal circumstances, where *C_f_*_1_ = *C_f_*_2_ = *C_f_*, *R_f_*_1_ = *R_f_*_2_ = *R_f_*, *C*_11_ = *C*_12_ = *C*_1_, *C*_21_ = *C*_22_ = *C*_2_, and the open-loop gain *A*(*s*) of these two amplifiers are identical, the output voltage can be estimated by

(1) $$u_{o}=-\frac{2I_{s}}{g_{f}}B\left(s\right)$$

where

(2) $$\begin{array}{l} B\left(s\right)=\frac{g_{f}Z_{s}}{g_{f}\left(2Z_{1}+Z_{s}\right)+\frac{1}{A\left(s\right)}\left(2+\left(g_{2}+g_{f}\right)\left(2Z_{1}+Z_{s}\right)\right)}\\ g_{f}=sC_{f},\;g_{1}=sC_{1},\;Z_{1}=1/g_{1},\;g_{2}=sC_{2} \end{array}$$

Note that *R_f_* is neglected due to its negligible current as compared with that of *C_f_*.

For a typical design, the transformer is symmetric. All the windings have the same number of turns, meaning *L*_1_ = *L*_2_ = *L*, *C_t_*_1_ = *C_t_*_2_ = *C_t_*, *R_L_*_1_ = *R_L_*_2_ = *R_L_*_3_ = *R_L_*. In this case, the current source *I_s_* and impedance *Z_s_* can be written as [25,27]

(3) $$\begin{array}{l} I_{s}=s\left(C_{a}-C_{b}\right)u_{i}\\ Z_{s}=\frac{Ls+R_{L}}{LC_{eq}s^{2}+R_{L}C_{eq}s+1} \end{array}$$

where *C_eq_* = 2*C*_0_ + 2*C_t_*. It can be noted that the reactive impedance *Z_s_* varies with frequencies. The transformer network impedance |*Z_s_*| and its real part Re(*Z_s_*) both get their maximum value simultaneously at the resonant frequency, where the imaginary part Im(*Z_s_*) is zero. The resonant angular frequency can be approximated by $\omega_{0}=1/\sqrt{LC_{eq}}$, assuming the quality factor *Q* is large enough (*Q* > 50), which is the case in the practical circuit. The quality factor *Q* is defended as following

(4) $$Q\left(\omega \right)=\frac{L\omega }{R_{L}}$$

At the resonate frequency, we have

(5) |Zs(ω0)|=Re(Zs(ω0))≈LQ0ω0

where *Q*_0_ = *Lω*_0_/*R_L_*.

We can assume that the open-loop gain of the amplifier is infinite, and then Equation (2) can be rewritten as

(6) $$B\left(s\right)\approx \frac{Z_{s}}{2Z_{1}+Z_{s}}$$

Combining this equation with Equations (2) and (3), we have

(7) $$B\left(s\right)\approx \frac{C_{1h}s\left(Ls+R_{L}\right)}{\left(C_{1h}+C_{eq}\right)s\left(Ls+R_{L}\right)+1}$$

where *C*_1*h*_ = *C*_1_/2. Since *C*_1_ is much larger than *C_eq_*, this equation becomes *B*(*s*) ≈ 1 for all angular frequencies over $\omega_{a}=\sqrt{2/LC_{1}}$ (*ω_a_* < *ω*_0_). In this case, the front-end circuit output voltage in Equation (1) can be written as

(8) $$u_{o}\approx -\frac{2I_{s}}{g_{f}}=-2u_{i}\frac{C_{a}-C_{b}}{C_{f}}$$

This implies that the output is similar to the injection sine wave, and the phase between them can be the same or the opposite. Its amplitude is proportional to the capacitance difference of the two capacitors under test. 

### 2.2. Noise Analysis

Although the front-end circuit is used to sensing the variations of the capacitance, the actual physical parameter it measures is the current. Therefore, the current noise of the circuit determines the capacitance resolution. In this section, we first focus on the current noise of the front-end circuit, and then discuss the signal-to-noise ratio and the capacitance resolution.

#### 2.2.1. The Equivalent Input Current Noise

**Figure 3:**
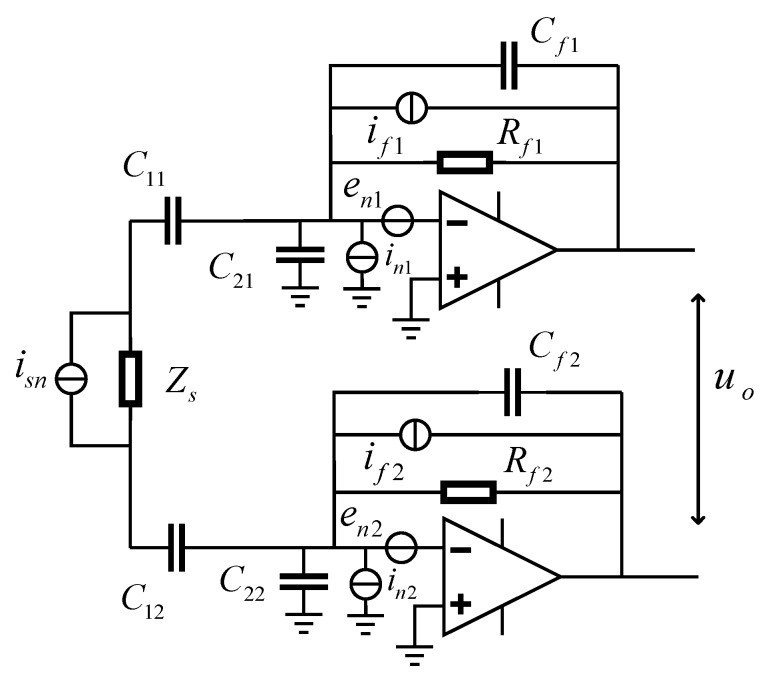
The schematic diagram of the front-end circuit for noise calculation.

The noise sources and their distributions are shown in Figure 3. To simplify the analysis, it can be assumed that the upper and lower half of the circuit presented in Figure 3 are identical. The thermal noise of the transformer network *i_sn_*, with a bandwidth of 1 Hz (similarly hereinafter), can be expressed by

(9) isn=4kBTRe(Zs)

where *k_B_* is the Boltzmann constant and *T* is the absolute temperature of the transformer. The current thermal noise of the feedback resistor *R_f_* is a white noise which is presented by $i_{f}=\sqrt{4k_{B}T/R_{f}}$. The noise of the amplifier is characterized by *e_n_* and *i_n_*, representing the input voltage noise and the input current noise, respectively. Since all of these noise sources are independent variables with random distributions, the total output voltage noise of the front-end circuit can be expressed as

(10) $$u_{o,n}^{2}={\left|\frac{2B\left(s\right)}{g_{f}}\right|}^{2}\left(i_{sn}^{2}+2{\left|\frac{e_{n}}{Z_{s}}D_{1}\left(s\right)\right|}^{2}+2{\left|\frac{i_{n}}{2}D_{2}\left(s\right)\right|}^{2}+2{\left|\frac{i_{f}}{2}D_{2}\left(s\right)\right|}^{2}\right)$$

where $D_{1}\left(s\right)=1+\frac{g_{2}+g_{f}}{2}\left(2Z_{1}+Z_{s}\right)$ and $D_{2}\left(s\right)=1+\frac{2Z_{1}}{Z_{s}}$.

Combining Equations (1) and (10), the signal-to-noise ratio (*SNR*) of the front-end circuit at its output port can be shown as

(11) $$SNR=\frac{{\left|u_{o}\right|}^{2}}{{\left|u_{o,n}\right|}^{2}}=\frac{I_{s,s}^{2}}{I_{s,n}^{2}}$$

where $I_{s,s}^{2}=\omega^{2}{\left(C_{a}-C_{b}\right)}^{2}U_{A}^{2}$, $I_{s,n}^{2}=i_{sn}^{2}+2{\left|\frac{e_{n}}{Z_{s}}D_{1}\left(s\right)\right|}^{2}+2{\left|\frac{i_{n}}{2}D_{2}\left(s\right)\right|}^{2}+2{\left|\frac{i_{f}}{2}D_{2}\left(s\right)\right|}^{2}$, *U_A_* is the amplitude of the injection sine wave. Additionally, *I_s,n_* can be regarded as the equivalent input noise of the front-end circuit at the node of *I_s_* in Figure 2b. It is apparent that the *SNR* is independent of the open-loop gain of the amplifier *A*(*s*). *D*_1_(*s*) and *D*_2_(*s*) can be extended as follows

(12) $$\begin{array}{l} D_{1}\left(s\right)=\left(1+\frac{C_{2fh}}{C_{1h}}\right)\frac{\left(Ls+R_{L}\right)\left(C_{eq}+C_{eq1}\right)s+1}{\left(Ls+R_{L}\right)C_{eq}+1}\\ D_{2}\left(s\right)=\frac{\left(C_{1h}+C_{eq}\right)\left(R_{L}+Ls\right)s+1}{C_{1h}\left(R_{L}+Ls\right)s} \end{array}$$

where $C_{2fh}=\frac{C_{2}+C_{f}}{2},\;C_{1h}=\frac{C_{1}}{2},\;C_{eq1}=\frac{C_{2fh}C_{1h}}{C_{2fh}+C_{1h}}$. In our practical circuit, *C*_1_ is in the order of 10^−5^ F, *C*_2_ and *C_f_* are in the order of 10^−11^ F, and *C_eq_* is in the order of 10^−9^ F. Considering the aforementioned conditions, *D*_1_(*s*) and *D*_2_(*s*) are approximately equal to one for frequencies greater than $1/\sqrt{L\left(C_{eq}+C_{1h}\right)}$. Thus, the equivalent input current noise becomes

(13) Is,n2≈isn2+2|enZs|2+in22+if22=4kBTRe(Zs)+2en2|Zs|2+in22+2kBTRf

It can be observed that the current noise of the front-end circuit is directly determined by the noise sources presented in Figure 3. 

*I_s,n_* sets the current resolution of the circuit in Figure 2 and needs to be minimized. Among the four terms in Equation (13), the amplifier current noise *i_n_* can be minimized only by choosing a low current noise op-amp component. Typically, junction field effect transistor (JFET) input amplifiers have a current noise as low as several fA/Hz^1/2^, which is much smaller than those of bipolar junction transistor (BJT) input amplifiers. Therefore, the amplifier with a JFET input stage is preferred. Additionally, *i_f_* is a white current noise derived from the feedback resistor, and is inversely proportional to the resistance. For a resistor of 100 MΩ, the current noise is 12.8 fA/Hz^1/2^ at room temperature. The feedback resistor current noise can be easily reduced by using a larger resistor. 

The remaining two terms are related to the impedance of the transformer network (i.e., *Z_s_*). Although the amplifier input voltage noise *e_n_* is typically a white noise over the kilohertz frequency, its contribution to the final equivalent current *I_s,n_* is frequency dependent, as the impedance *Z_s_* varies with the frequency. In Section 2.1, it was shown that the impedance *Z_s_* and its real part Re*(Z_s_*) reach their maximum values at *ω*_0_, which are both equal to *LQω*_0_. Therefore, the equivalent input current noise of the front-end circuit can reach its minimum value, which can be expressed by

(14) $$I_{s,n,\min}^{2}\approx \frac{4k_{B}T}{LQ\omega_{0}}+\frac{2e_{n}^{2}}{{\left(LQ\omega_{0}\right)}^{2}}+\frac{i_{n}^{2}}{2}+\frac{2k_{B}T}{R_{f}}$$

Among the four terms in Equation (14), the terms $\frac{2e_{n}^{2}}{{\left({LQ\omega }_{0}\right)}^{2}}$ and $\frac{2k_{B}T}{R_{f}}$ are negligible. The reasoning is provided as follows. Parameters for a differential transformer with *L* = 4.2 mH and *Q* = 400 at the resonant frequency of 100 kHz were adopted from Reference [18] for later analysis. With these parameters, the impedance at 100 kHz can be estimated as 1.1 MΩ, which will generate a current noise of 122.7 fA/Hz^1/2^ by the transformer. The current noise *i_n_* of a low-noise amplifier is on a similar order of magnitude. In comparison, a low-noise amplifier usually has a voltage noise *e_n_* in the order of 10 nV/Hz^1/2^, so the current noise contributed by *e_n_* would be smaller than 10 fA/Hz^1/2^ according to Equation (14). Additionally, as mentioned previously, for a resistor of 100 MΩ, the current noise is 12.8 fA/Hz^1/2^. These two noise sources are one order of magnitude smaller compared with the other two terms. Therefore, the transformer thermal current noise and the amplifier current noise are likely to dominate the noise level of the front-end circuit in Figure 2. Thus, Equation (14) can be reduced to

(15) $$I_{s,n,\min}^{2}\approx \frac{4k_{B}T}{LQ\omega_{0}}+\frac{i_{n}^{2}}{2}$$

It can be observed that the input-referred current noise decreases as *ω*_0_ increases and a large *ω*_0_ can be achieved through tuning the capacitor *C_t_*.

Two parameters within the equation, inductance *L* and quality factor *Q*, are related to the transformer, and it is the *LQ* product that ultimately determines the final noise *I_s,n,min_*. A high *LQ* product is critical for the noise optimization. For a given differential transformer, the capacitors in the front-end circuit in Figure 2 have negligible effects on the *LQ* product, which is predominantly determined by the transformer design. Therefore, the *LQ* product is chosen as the figure-of-merit for the differential transformer design discussed below. This parameter can also be used to compare transformers with different inductance values or quality factors.

#### 2.2.2. Capacitance Resolution

Since capacitance needs to be measured with the front-end circuit, the resolution of the circuit in terms of capacitance must be discussed. By definition, the signal-to-noise ratio of the circuit is

(16) $$SNR={\left(\frac{C_{s,s}}{C_{s,n}}\right)}^{2}$$

where *C_s,s_* = *C_a_* − *C_b_*, *C_a_* and *C_b_* are the capacitors under test shown in Figure 2, and *C_s,n_* is the capacitance noise of the circuit. Combine this with Equation (11) and we obtain

(17) $$C_{s,n}=\frac{I_{s,n}}{\omega U_{A}}$$

where $U_{A}$ is the amplitude of the injection sine wave. Since $U_{A}$ is a constant that has no effect on *I_s,n_*, increasing $U_{A}$ is an effective approach to reduce capacitance sensing noise. For the capacitance noise minimization, the conventional procedure is to set the derivative of *C_s,n_* equal to zero. Therefore, we can obtain the expression below

(18) $$I_{s,n}\left(\omega \right)-\omega I_{s,n}^{\prime }\left(\omega \right)=0$$

where $I_{s,n}^{\prime }\left(\omega \right)$ is the derivative of $I_{s,n}\left(\omega \right)$ with respect to the frequency *ω*. Since $I_{s,n}^{\prime }\left(\omega_{0}\right)=0$ and $I_{s,n}\left(\omega_{0}\right)=I_{s,n,\min}\approx 0$, the resonant frequency *ω*_0_ could be considered as an approximate root of Equation (18). This means that at the resonant frequency *ω*_0_, an excellent capacitance resolution can be achieved. Thus, for the capacitance resolution optimization, the frequency of the injection sine wave is ideally *ω*_0_. The minimum capacitance noise can then be expressed as

(19) $$C_{s,n,\min}\approx \frac{I_{s,n,\min}}{\omega_{0}U_{A}}$$

Rearranging Equation (19), one can obtain

(20) $$C_{s,n,\min}U_{A}\approx \frac{I_{s,n,\min}}{\omega_{0}}$$

Here, we define a noise figure-of-merit (FOM) for the electronics, which is determined by the resonant frequency and the noise sources from the transformer and the amplifier. In the definition of FOM, the factor of injection wave amplitude $U_{A}$ is excluded, so the FOM reflects only the noise performance of the front-end electronics. It can be used in future work to optimize the capacitance resolution of the circuit and fully capitalize the transformer and the low-noise amplifier.

## 3. Experiment Results and Discussion

To achieve front-end electronics with a low noise, special attention was paid to design and fabricate a transformer with a high *LQ* product, which could remain in the relatively high frequency band. Various types of transformer cores and winding methods were carefully investigated in the fabrication process. We collected a large amount of data to characterize their influence on the *LQ* product. Some basic parameters of the final transformer are listed in Table 2.

**Figure 4:**
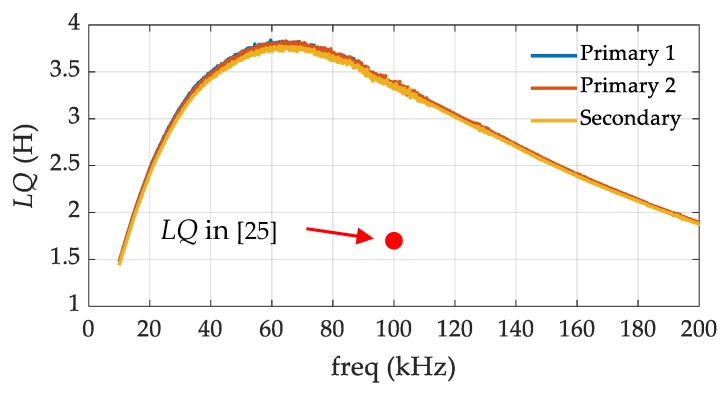
Measured *LQ* product of our transformer and the *LQ* production in Reference [25].

**Figure 5:**
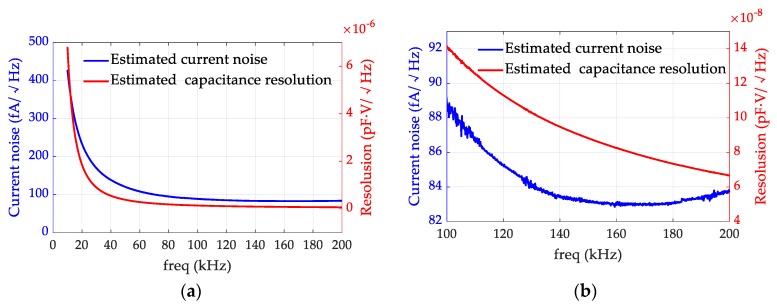
Minimum current noise and capacitance resolution of the front-end circuit limited by the transformer. (**a**) Noises from 10 kHz to 200 kHz. (**b**) More details of the curves in the frequency band between 100 kHz and 200 kHz. The blue curves represent the minimum current noise calculated with Equation (14). The red curves represent the minimum capacitance resolution calculated with Equation (20).

An impedance analyzer, Keysight E4990A (Keysight Technologies, Santa Rosa, CA, USA), was used to measure and analyze the performance of the transformer. Frequency was swept in the range of 10−200 kHz, and the inductance and the quality factor were measured in *Ls-Q* mode. Two primary windings and one secondary winding were characterized individually. Their respective *LQ* products were calculated based on the experimental data over the frequency band, and the results are shown in Figure 4. The three curves agree well with each other, indicating that the windings’ parameters are within tolerance. Due to the similar parameters, the curve of the secondary winding is examined here without loss of generality. The *LQ* product of the winding is larger than 1.5 H over the entire frequency band shown in the figure. From 10 kHz to 40 kHz, the *LQ* product increases as the frequency increases, with a slope of approximately +75.6 mH/kHz. For frequencies above 100 kHz, the curve decreases with a constant slope of −15.5 mH/kHz. In the band of 40−100 kHz, the slope of the curve turns from a positive value to a negative value, and the *LQ* product reaches its maximum value of 3.82 H at the frequency of approximately 65 kHz. Furthermore, Figure 5 indicates that the resistive resistance (*R*) increases at a higher rate above 40 kHz than the increase of the reactive resistance (*Lω*), due to the losses of the transformer core and windings. It should be pointed out that the *LQ* product at 100 kHz is 3.36 H, which is roughly two times higher than that in Reference [25] (i.e., 1.7 H), representing an improvement in the figure-of-merit of the transformer. 

Assuming the amplifiers and the feedback resistors have negligible noises, the current noise and the capacitance resolution of the front-end circuit would be constrained by the thermal noise of the transformer. Using Equations (14) and (20), the fundamental limits of the noise performance can be estimated from the experimental data in Figure 4. The results are shown in Figure 5. It can be observed that at 10 kHz, the current noise and capacitance resolution resulting from the transformer noise are very high, but they both decrease with the increasing frequency. Even though the *LQ* curve reaches its maximum value at 65 kHz, neither the current noise (blue line) nor the capacitance resolution (red line) reaches a minimum value. It can be concluded from these estimations that the frequency, where the maximum *LQ* product was located, was not necessarily the optimal operating frequency for the front-end circuit in our study. Figure 5b shows the results in the frequency band between 100 kHz and 200 kHz in detail. In this band, the capacitance resolution monotonically decreases while the current noise curve reaches its minimum value around 160 kHz and then rises slightly. The reason for this can be explained by Equations (11) and (19). Although the equivalent noise is almost flat as the frequency increases, the signal is always proportional to the frequency, so the *SNR* becomes larger and the capacitance resolution becomes better in higher frequency bands.

Table 3 lists some values at some typical frequencies in Figure 5. The capacitance resolution at 200 kHz is 0.067 aF·V/Hz^1/2^, which is about five times and two times lower than the values at 50 kHz and 100 kHz, respectively. This indicates that the resolution frequency of the transformer network should be set as high as possible. Assuming the amplitude of the injection sine wave, *U_A_*, is 0.6 V, the capacitance resolution of the front-end circuit can be estimated to be 0.11 aF/Hz^1/2^. Table 4 lists some capacitance resolution results from existing work. Our work shows a better capacitance resolution due to a higher *LQ* product and a higher resonant frequency.

## 4. Conclusions

In order to find methods for the noise performance optimization, the capacitance sensing front-end circuit was modeled and analyzed in this paper. The analysis showed that the *LQ* product and *I*_*s*,*n*,min_/*ω*_0_ can be used to characterize the transformer and the capacitance resolution, respectively. Enlarging the *LQ* product and the resonant frequency of the transformer network would be the effective way to lower the capacitance sensing noise. The high *LQ* transformer provided in the article would generate a capacitance noise of 0.067 aF·V/Hz^1/2^ at the resonant frequency of 200 kHz, which is equivalent to a capacitance resolution of 0.11 aF/Hz^1/2^ with an amplitude of 0.6 V of the injection wave. Therefore, the transformer could satisfy the requirements of the TianQin mission and reduce the pressure on other modules in the system.

## Figures and Tables

**Table 1:** Comparison of typical parameters for the mission of laser interferometer space antenna (LISA) [8,24,25] and TianQin [19].

Items	LISA	TianQin
Orbit center	Sun	Earth
Arm length	2.5 × 10^6^ km	1.7 × 10^5^ km
Number of spacecrafts	3	3
Residual acceleration	3 × 10^−15^ ms^−2^/Hz^1/2^ @1 mHz	10^−15^ ms^−2^/Hz^1/2^ @6 mHz
Cubic test mass	46 × 46 × 46 mm^3^	50 × 50 × 50 mm^3^
Weight of test mass	2 kg	2.5 kg
Electrode gap for *x*-axis	4 mm	5 mm
Capacitance of single frame electrode for *x*-axis	1.15 pF	1.4 pF
Amplitude of injection voltage	0.6 V	1.4 V
Capacitance resolution	1 aF/Hz^1/2^	≤0.69 aF/Hz^1/2^
Position sensing accuracy	1.8 nm/Hz^1/2^	≤1.7 nm/Hz^1/2^
Electrostatic stiffness	0.25 × 10^−7^/s^2^	0.92 × 10^−7^/s^2^

**Table 2:** Basic parameters of the differential transformer.

Parameter	Typical Value
Transformer inductance	8.2 mH
Self-resonant frequency	463 kHz
Stray capacitance	14 pF
DC resistance	1.8 Ω
Coupling factor	0.98

**Table 3:** Values at typical frequencies.

Frequency (kHz)	*LQ* (H)	Estimated Current Noise (fA/Hz^1/2^)	Estimated Capacitance Resolution (aF·V/Hz^1/2^)
50	3.64	120.3	0.38
65	3.82	103.0	0.25
100	3.36	88.6	0.14
160	2.38	82.7	0.082
200	1.88	83.7	0.067

**Table 4:** Noise performance comparison with existing work.

Existing Work	Resonant Frequency (kHz)	Amplitude of Injection Voltage (V)	Capacitance Resolution (aF/Hz^1/2^)
P. Touboul et al. [28]	100	7.1	0.1
J.P. Marque et al. [29]	100	7.6	0.2
M. Armano et al. [16]	100	0.6	0.7
M. Hu et al. [14]	50	8.5	0.14
This work	200	0.6	0.11
